# Selenium and Metabolic Disorders: An Emphasis on Type 2 Diabetes Risk

**DOI:** 10.3390/nu8020080

**Published:** 2016-02-06

**Authors:** Ashley N. Ogawa-Wong, Marla J. Berry, Lucia A. Seale

**Affiliations:** Department of Cell and Molecular Biology, John A. Burns School of Medicine, University of Hawaii, 651 Ilalo St., Honolulu, HI 96813, USA; mberry@hawaii.edu (M.J.B.); lseale@hawaii.edu (L.A.S.)

**Keywords:** selenium, selenoproteins, metabolic disease, trace element

## Abstract

Selenium (Se) is a micronutrient that maintains biological functions through the action of Se containing proteins known as selenoproteins. Due to the known antioxidant effects of Se, supplements containing Se have been on the rise. While Se supplementation may be beneficial for Se deficient populations, few are at risk for Se deficiency due to the transportation of food from Se-rich regions and the rise of Se-enriched foods. Alarmingly, Se supplementation may have adverse effects in people who already receive an adequate Se supply. Specifically, an increased risk of type 2 diabetes has been reported in individuals with high baseline Se levels. However, this effect was restricted to males, suggesting the relationship between Se and glucose homeostasis may be sexually dimorphic. This review will discuss the current understanding of the interaction between Se and glucose homeostasis, including any sex differences that have been described.

## 1. Introduction

Dietary Selenium (Se) is critical for the synthesis of selenoproteins, which carry out the biological functions of Se. To date, 24 murine and 25 human selenoprotein genes have been identified [1]. Of these gene products, the glutathione peroxidases and thioredoxin reductases, which participate in redox reactions, are likely the most well-studied selenoprotein families. Other notable selenoproteins with known functions include the iodothyronine family, which regulate thyroid hormone activation and Selenoprotein P (Sepp1), which is necessary for Se transport through the serum. Thus, selenoproteins function in a wide variety of processes (Table 1). Sources rich in Se include seafood, organ meats, dairy, grain, cereals, and Brazil nuts, albeit Se concentrations in plants are dependent on the Se levels in the soil, and the plant’s capacity to uptake Se. Thus, populations that live in areas with low soil Se are at risk for Se deficiency. Complications from Se deficiency include Keshan disease [2], a cardiomyopathy, and Kashin-Beck disease [3], an osteocondrapathy. Low Se levels have also been implicated in male infertility [4]. The current daily recommended value for adults is 55 µg/day in the United States, a value which was determined based on maximal glutathione peroxidase activity [5]. The dose range for Se that is beneficial for human health is fairly narrow, and has been described as a U-shaped curve.

**Table 1:** List of identified mammalian selenoprotein genes and their known functions.

Selenoprotein Gene	Abbreviation	Function(s)
15 kDa-selenoprotein	Sep15	Protein folding
Iodothrionine Deiodinase 1–3	Dio1–3	Thyroid hormone activity regulation
Glutathione Peroxidase 1–4, 6	GPx1–6	Hydroperoxide/phospholipid peroxide reduction
Methionine-R-Sulfoxide Reductase 1	MsrB1	Reduces oxidized methionine residues
Selenoprotein H	SelH	Genome maintenace
Selenoprotein I	SelI	Unknown
Selenoprotein K	SelK	ER-associated degradation; inflammation
Selenoprotein M	SelM	Ca^2+^ homeostasis
Selenoprotein N	SelN	Muscle development
Selenoprotein O	SelO	Unknown
Selenoprotein P	Sepp1	Selenium transport
Selenoprotein S	SelS	ER-associated degradation; inflammation
Selenophosphate Synthase 2	SPS2	Selenoprotein biosynthesis
Selenoprotein T	SelT	Ca^2+^ homeostasis; neuroendocrine secretion
Thioredoxin Reducase 1–3	TrxR1–3	Disulfide bond reduction
Selenoprotein V	SelV	Unknown
Selenoprotein W	SelW	Unknown

Se was long touted for its cytoprotective properties, due to its ability to upregulate antioxidant selenoenzymes. Thus, it was believed that Se supplementation could prevent the onset of metabolic diseases, such as type 2 diabetes (T2D), by counteracting oxidative stress. Indeed, Se in the form of selenate, was found to act as an insulin mimetic, displaying anti-diabetic effects [6]. In support of this, two cross-sectional studies reported lower baseline Se levels to be associated with T2D incidence among elderly French men [7], as well as in samples taken from a population in southeastern Spain [8]. A more recent, longitudinal study conducted in the United States, reported higher toenail Se to be associated with lower T2D risk [9]. However, other cross-sectional studies, namely the National Health and Nutrition Examination Survey (NHANES) III [10] and NHANES 2003–2004 [11], revealed an association between high Se intake and metabolic disease. Moreover, increased T2D risk was found to be a secondary outcome in the Nutritional Prevention of Cancer (NPC) trial, a randomized, controlled trial assessing the efficacy of Se supplementation in the form of Se yeast (200 µg/day) in preventing skin cancer [12]. The Selenium and Vitamin E Cancer Prevention Trial (SELECT), testing the effects of selenomethionine (SMet, 200 µg/day) and/or Vitamin E in preventing prostate cancer was curtailed as it became apparent Se supplementation did not appear beneficial in the prevention of prostate cancer, and a nonsignificant trend towards T2D in the experimental group was reported [13,14]. Yet, other epidemiological studies and clinical trials failed to find a correlation between increased Se and T2D susceptibility [15]. One reason for the discrepancies in the human trials may be due to differences in baseline Se levels. For instance, the mean baseline Se levels in SELECT [13] subjects were already high (136 µg/L) whereas only the NPC [12] subjects in the upper third tertile of baseline Se (>122 µg/L) demonstrated higher incidence of T2D. Strengthening the idea of a narrow beneficial window of Se dose, it is likely that with regards to T2D, Se supplementation may be advantageous in populations with low Se status, but detrimental in Se-replete populations. In fact, randomized, controlled trials of Se supplementation in elderly patients [16] and pregnant women [17] from the UK, who have lower baseline Se than US subjects, did not result in increased T2D risk, as determined by serum adiponectin concentration. Another source of the inconsistencies might be attributable to differences in Se source. As discussed in detail below, different Se forms vary in their bioavailability and biological effects. Thus, it is difficult to delineate a clear-cut relationship between Se status and T2D based on evidence from the current human clinical trials and epidemiological studies.

To obtain a clearer picture of the role of Se in metabolic disease, this review will focus on the current understanding of the connections between dietary Se, selenoproteins, and energy metabolism, with an emphasis on animal models. Particularly interesting is that the relationship between Se metabolism and glucose homeostasis appears to be sexually dimorphic, a topic that will also be discussed later in this review.

## 2. Forms of Selenium

Selenium occurs ubiquitously in the environment, but its biological activity is determined by the form that reaches an organism and how this form is metabolized. In general, the Se content of plants reflects the Se content of the surrounding soil and its bioavailability, and crop and livestock Se content will also reflect the Se content of the soil and the forage items they ingest [18]. Besides the dependence on the soil content, plant Se content may also vary according to pH and the presence of soil ions that can form complexes with Se, enhancing or decreasing its bioavailability, according to the bacterial species present in the roots, and according to the ability of plants to uptake, accumulate and metabolize Se in its various forms [19,20,21].

During amino acid synthesis, plants generally employ Se and sulfur nonspecifically in their metabolic processes. Thus, most plants form methionine (Met) and SeMet in amounts that reflect the relative sulfur and Se concentrations of the soils in which they are grown [22]. The metabolic processes of SeMet and its downstream metabolites are generally analogous to those of Met in both plants and animals. Nevertheless, once incorporated in animal proteins in place of Met, the Se of SeMet can become part of an unregulated pool of Se, or be released when the amino acid is metabolized via methionine cycle or transsulfuration pathways, becoming part of the highly regulated selenocysteine (Sec) pool [23].

Efficient uptake and metabolism of dietary Se in animals will depend on which chemical form was ingested. Predominant forms of inorganic Se are selenite and selenate, both water-soluble [24]. Organic forms of Se mostly include the amino acids SeMet and Sec [25], and rare organic forms such as selenoneine, Se-methylselenocysteine, and selenoglutathione may have important biological roles that are currently unknown [20,26]. Inorganic selenite is absorbed by the enterocytes at rates that vary from 50% to 90% [23,27,28], depending on age, sex, and dietary constituents. However, the molecular mechanism responsible for Se absorption is poorly understood. Gastrointestinal selenate absorption is conducted transcellularly, possibly by the SLC26 multifunctional anion exchanger family, and paracellularly through the intestinal tract with virtually 100% efficiency [28,29,30,31]. Nevertheless, it is necessary for selenate to be reduced to selenite prior to being metabolized, and thus these inorganic forms possess lower retention rates when compared to organic forms [29,30,32]. In the case of organic SeMet, more than 90% is uptaken transcellularly by enterocytes [27,33]. Although the available data for Se absorption is limited, the European Safety Food Authority recently stated the Se absorption efficiency on a normal diet to be ~70% [34]. However, the United States National Institutes of Health guidelines does not state an overall absorption value for Se, due to the discrepancies in absorption rates of different Se forms [5].

Physiological to low-toxic doses of Se are known to be excreted as methylated sugar metabolites, specifically 1β-methylseleno-*N*-acetyl-d-galactosamine in the urine, while toxic levels of Se lead to the excretion of trimethylselenonium ion and dimethylselenide in the urine and breath [35].

The metabolic fates of organic and inorganic forms point to their conversion intracellularly to selenide to be utilized in selenoprotein synthesis [27,36]. Neither Sec nor SeMet accumulate in biological systems, Sec being promptly converted into selenide via decomposition of Sec by the enzyme Sec β-lyase (Scly), and SeMet via demethylation to methylselenol via γ-lyase activity [23,37,38].

Thio- and seleno-amino acids are structurally and metabolically analogous, differing in having either sulfur or selenium bound to the γ-carbon of their side chains. Nevertheless, they differ in physiological abundance, reactivity, acid dissociation constant (pKa) and metabolic roles. Met and SeMet are incorporated into proteins and peptides nonspecifically in amounts that reflect their tissue abundance. SeMet is broken down into methylselenol by the tetrameric enzyme cystathione γ-lyase (Cth), a member of the reverse transsulfuration pathway [39,40]. This pathway converts homocysteine to cysteine (Cys), which contributes to the generation of sulfide [41]. The methylselenol generated in the Cth-catalyzed reaction of SeMet can be demethylated and utilized in selenoprotein biosynthesis, including for the synthesis of GPx1 [42]. Because SeMet can contribute to the Se pool for selenoprotein biosynthesis, it is considered an additional source of biologically essential Se. Sec and Cys have analogous molecular structures, but Sec is exclusively inserted into genetically unique selenoprotein families, requiring a recoding of the UGA stop codon that will be further explored below. Interestingly, it has been uncovered that Cys can be incorporated in place of Sec in the enzymes thioredoxin reductase 1 (TrxR1) and 3 (TrxR3), using the Sec synthesis machinery. Cys incorporation encoded by UGA is enhanced in conditions of low dietary Se, suggesting a mechanism to reduce selenoprotein activity [43].

## 3. Selenoprotein Synthesis

Selenoprotein synthesis involves several processes that are distinct from normal protein translation. Eukaryotic Sec biosynthesis is a unique process beginning with the synthesis of Sec directly on its tRNA (Sec-tRNA[Ser]Sec) [44], requiring the aminoacylation of tRNA[Ser]Sec with serine via the action of seryl-tRNA synthetase. Phosphoseryl-tRNA kinase (PSTK) phosphorylates the resulting seryl-tRNA[Sec]Ser to form phosphoseryl-tRNA[Ser]Sec [45]. In a separate reaction, selenide is activated by selenophosphate synthetase 2 (SPS2), yielding selenophosphate [46,47]. The final step involves selenophosphate transfer onto phosphoseryl-tRNA[Ser]Sec by selenocysteine synthase (SecS), generating Sec-tRNA[Ser]Sec [48].

Perhaps one the most intriguing aspects of selenoprotein biosynthesis is the ability of Sec-tRNA[Sec]Ser to recognize the UGA codon, allowing for the recoding of what was conventionally thought to serve solely as a stop codon into a Sec insertion site. Consequently, intricate mechanisms have evolved to prevent premature translation termination. Sec insertion is dependent on the presence of a secondary structure known as the Sec Insertion Sequence (SECIS) element in the 3′ untranslated region of the selenoprotein mRNA [49]. The SECIS interacts with SECIS binding protein 2 (SBP2) [50,51], allowing for the recruitment of Sec-tRNA[Ser]Sec and Sec-specific elongation factor (EFSec) to the ribosome [52,53]. Other trans-acting factors have also been identified, including ribosomal protein L30 [54,55], SECp43, and SecS [56,57].

The presence of Se regulates selenoprotein synthesis. It was recently demonstrated through ribosome profiling that Se influence over selenoprotein synthesis occurs mainly through changes in translational efficiency [58]. Reduced Sec incorporation causes the UGA codon to be read as a premature stop codon, activating the nonsense-mediated decay (NMD) pathway [59]. However, it is important to note that selenoproteins are not affected equally by this pathway. Housekeeping selenoproteins such as TrxR1 and glutathione peroxidase 4 (GPx4) are more resistant to changes in Se levels, whereas the stress-response selenoproteins are more reactive to fluctuating Se status.

Sec decomposition occurs through Scly, a non-selenoenzyme that was initially purified from pig liver [60]. Scly has the ability to specifically distinguish Sec from the structurally similar amino acid, Cys [61]. In the presence of cofactor pyridoxal 5-phosphate, Scly cleaves Sec to form alanine and selenide. It was proposed that the selenide produced via Sec decomposition can be re-purposed for selenoprotein synthesis, implicating a role for Scly as a Se recycling enzyme when Se supply is low. In support of this, Scly was found to be required for selenoprotein biosynthesis *in vitro* when Sec is acting as the Se source [62]. Sec can be produced from selenoprotein degradation or alternatively generated by the transsulfuration pathway from selenomethionine [38]. However, Scly depletion did not hinder selenoprotein biosynthesis *in vitro* when selenomethionine was the sole source of Se [62], suggesting selenomethionine is able to contribute to the Sec pool through an alternate pathway. Moreover, Sec was found to donate Se to SPS to form selenophosphate, leading to the hypothesis that Scly and SPS enzymes work in a complex. *In vitro* immunoprecipitation studies confirmed Scly interaction with SPS1 and 2 [63]. The Se transport protein Sepp1, which contains multiple Sec residues, is synthesized primarily in the liver and secreted into the serum where it can be delivered to various tissues depending on need. Mice lacking both Scly and Sepp1 develop severe neurological deficits, surpassing the neurological phenotypes observed when only one gene is absent [64]. Hence, it has been suggested that Scly and Sepp1 act in tandem, with Sepp1 delivering Sec to tissues where Scly can cleave and recycle Se. Overall, the Sec decomposition pathway may also serve as a Se recycling pathway.

## 4. Selenium and Metabolic Disease

Although the association between Se supplementation and T2D in humans is considered to be controversial, studies in animal models may provide insights into some of the inconsistencies reported in human clinical trials. In a study comparing three different concentrations of Se in the diet, it was found that mice on a 0.4 ppm selenite diet developed insulin resistance, a hallmark of T2D [65]. This concentration of Se is comparable to the 200 µg Se regimen that was administered to humans in the NPC trial. High Se exposure also led to insulin resistance in rats, which was attributed to both excessive ROS production and attenuated ROS [66]. Moreover, 16 weeks of Se supplementation in pigs on an already Se adequate diet resulted in a trend towards increased body weight and HOMA-IR score, a measure of insulin resistance [67]. This was accompanied by alterations in glucose and lipid metabolic pathways in insulin-sensitive tissues. With the exception of the skeletal muscle, glutathione peroxidase activity and thioredoxin reductase activities were largely unchanged in insulin-sensitive tissues in response to Se supplementation, suggesting selenoprotein expression was already saturated under Se adequate conditions. These results suggest the proclivity towards metabolic disease in pigs receiving supranutritional Se doses may be a result of nonspecific incorporation of SeMet, rather than a consequence of increased selenoprotein activity. It is important to note that most of the parameters measured in this study only trended towards significance, and thus it was concluded that supranutritional Se contributes to but does not cause T2D. However, as the duration of the study was relatively short, it is premature to speculate whether significance would have been achieved under long-term supplementation.

*In vitro* studies in pancreatic islets and the mouse insulinoma derived β-cell line, MIN6, have demonstrated increased insulin content and secretion in response to Se treatment, leading to the hypothesis that Se is protective in the endocrine pancreas [68]. Higher levels of plasma Se were indeed found to be associated with elevated serum insulin in mice [65], but it is not known whether this is protective or detrimental. However, selenate treatment conferred protection to rat insulinoma cells, INS1, against streptozotocin-induced β-cell death [69], suggesting the antioxidant properties of Se may be protective in the late stages of T2D.

The link between inflammation and T2D is well-documented. Briefly, pro-inflammatory cytokines, including Interleukin (IL)-1β, IL-6, and tumor necrosis factor (TNF)α, are elevated in T2D subjects, where they have been shown to promote insulin resistance in the adipose tissue, liver, and muscle, and β-cell failure in the pancreas [70]. The expression of these pro-inflammatory cytokines is dependent on the nuclear translocation of the transcription factor, nuclear factor-kappa B (NF-κB). As mentioned above, Se reportedly plays a physiological role in inflammation [71]. Notably, Se was found to inhibit the NF-κB pathway [72,73]. It is therefore conceivable that inflammation may link Se to T2D. In fact, selenite was able to rescue blood glucose levels of streptozotocin-induced diabetic rats [74]. In selenite-treated diabetic rats, hepatic NF-κB expression was found to be reduced, likely contributing to the improved phenotype. However, the necessity of the reduction in hepatic NF-κB expression in selenite-mediated reversal of streptozotocin effects were not tested. In a separate study, pharmacological doses of selenite administered to streptozotocin-induced diabetic mice partially rescued blood glucose levels [75]. Selenite-treated mice exhibited lower levels of pancreatic pro-inflammatory cytokines (IL-1β, TNF-α, and interferon (INF)-γ) and lipid peroxidation.

These studies demonstrate that both oversupplementation and deficiency of Se can be associated with T2D risk, following a U-shaped curve. It appears that Se oversupplementation may promote T2D in an otherwise healthy animal. However, in diabetic animals which may have suboptimal Se status, Se appears to have beneficial effects by preventing further development of T2D complications. Additional human studies will be useful in determining the appropriateness of Se supplementation with respect to T2D.

## 5. Selenoproteins in Metabolic Disease

Selenoproteins are important metabolites of dietary Se, fulfilling the catalytic effects of Se. Thus, in order to clarify the discrepancies in the association between Se supplementation and metabolic disease, it is necessary to understand the role of each selenoprotein in maintaining glucose homeostasis. This section will review what is currently known about the roles of selenoproteins that have been connected to metabolic disease.

### 5.1. Glutathione Peroxidase 1

Glutathione Peroxidase 1 (GPx1) is the primary cytosolic peroxide scavenger, reducing peroxides to water, and protecting the cell from free radical damage. Considered to be a stress responsive selenoprotein, GPx1 expression is sensitive to Se intake. Overexpression of GPx1 in mice yielded surprising results, leading to reduced glucose clearance, hyperinsulinemia, hyperglycemia, and diminished insulin signaling [76]. Diet restriction alleviated all metabolic symptoms except hyperinsulinemia, suggesting the functional role of GPx1 lies in regulating insulin production [77]. Indeed, it was found that the H3 and H4 histones in the proximal promoter region of the insulin gene transcription factor, pancreatic duodenal homeobox-1 (PDX1), were hyperacetylated, ultimately leading to hyperinsulinemia. Although not shown directly, the hyperacetylation was speculated to be due to enhanced H_2_O_2_ scavenging. The importance of H_2_O_2_ in cellular signaling was established further when it was demonstrated that GPx1 null mice have increased insulin sensitivity in the muscle, resulting in a high fat diet-resistant phenotype [78]. The absence of GPx1 allowed for the oxidation of phosphatase and tensin homolog (PTEN), a member of the PTP family. Oxidation of PTEN inhibits its activity, sensitizing insulin signaling.

In contrast, several studies suggest that GPx1 might play a protective role against T2D. GPx1 overexpression in HIT-T15 cells protects against β-cell dysfunction induced by ribose treatment [79]. As ribose was found to induce oxidative stress in human islets, it is possible that GPx1 promotes β-cell survival through its ability to scavenge peroxides. Although constitutive GPx1-/- mice appear to preserve insulin signaling in response to an obesogenic diet, in the context of insulin secretion, GPx1 deficiency can be detrimental [80]. In the absence of GPx1, excess ROS in the pancreatic islets oxidize PTPN2, promoting STAT1 signaling which results in downregulating key enzymes of the insulin production and secretory pathway, such as Pdx1. Inactivation of hepatic PTPs due to excess ROS also appears to promote obesity and T2D disease progression. In hepatocytes isolated from GPx1-/- mice, the presence of excess ROS inactivates PTPN2, which negatively regulates STAT5-induced lipid synthesis [81]. Thus, hepatic GPx1 may prevent hepatic steatosis indirectly by regulating ROS levels. Further supporting the protective function of GPx1, the GPx mimetic, ebselen, was found to restore islet function in GPx1-/- mice through a PGC-1α dependent mechanism [82]. Nrf2 is a transcription factor that controls the transcription of antioxidant enzymes by interacting with an antioxidant response element (ARE). Several selenoproteins and selenoprotein synthesis factors were found to contain an ARE in their promoters, and are thus Nrf2-responsive. GPx1 levels were found to be suppressed when mice were fed a high fat diet due to 12-lipoxygenase activity, which was found to inhibit Nrf2 nuclear translocation [83]. Pancreatic islet specific 12-lipoxygenase deletion resulted in GPx1 upregulation in response to high fat feeding. The resulting reduction in oxidative stress was found to be beneficial in preserving islet β-cell function under high fat diet consumption in mice. To corroborate these studies, a genetic polymorphism in the GPx1 gene which results in lower GPx activity, was found to correlate with increased incidence of metabolic syndrome in a cohort of Japanese men [84].

Generally speaking, both GPx1 overexpression and deficiency appear to have negative effects in metabolic disease. These findings are aligned with the U-shaped therapeutic dose effect of Se intake. However, the function of GPx1 is tissue dependent, exhibited by the differences in outcome of GPx1 deficiency in the muscle [78], liver [81], and pancreatic islets [80]. Increased understanding of tissue-specific functions of GPx1 will improve our knowledge of the role GPx1 plays in metabolic disease. It is also important to note the GPx1 response may also have a timing specific component in that excess GPx1 in the pre-disease state may promote disease pathogenesis. The disease state, however, may suppress GPx1 expression, thus, targeting GPx1 pathways may be beneficial.

### 5.2. Selenoprotein P

In humans, a positive correlation between hepatic SEPP1 expression and T2D has been reported [85]. In this same study, increased hepatic SEPP1 mRNA expression was also associated with reduced glucose tolerance and higher fasting glucose levels, which are indicative of insulin resistance. However, it is important to note that serum Sepp1 levels become saturated at high Se intake [86]. One limitation of this study [85] is that the patients’ Se intake levels were not reported. Likely, the usefulness of Sepp1 as a biomarker is limited to subjects who do not receive an optimal Se intake. Additionally, since Se deficiency has been reported in T2D patients [7], it is possible that Sepp1 mRNA expression is elevated in the diseased state, as Se transport will be in higher demand. Thus, the elevation of Sepp1 may be a secondary effect of T2D, rather than a cause. Nevertheless, adiponectin, an adipokine with anti-diabetic effects [87], was found to be inversely correlated with serum Sepp1 levels in T2D patients [88]. Moreover, elevated serum Sepp1 levels were positively correlated with carotid intima-media thickness and C-reactive protein, both of which are predictors for cardiometabolic disease [89]. Studies of SEPP1 genetic variants reveal SEPP1 polymorphisms to be associated with fasting insulin and the acute insulin response [90]. Taken together, Sepp1 appears to be involved in glucose homeostasis, although direct conclusions cannot be made due to the correlative nature of these studies. Mouse models and cell lines have been used to delineate the mechanistic relationship between Sepp1 and carbohydrate metabolism. For example, studies in HepG2 cells revealed that hepatic Sepp1 mRNA and promoter activity are under the control of insulin and a supramolecular complex composed of PGC1α, FoxO1a, and HNF-4α, which also regulates gluconeogenic enzymes PEPCK and G6Pase [91]. Additionally, Sepp1 was found to negatively regulate insulin signaling in the liver, through AMPK inactivation in female mice [85]. Sepp1 also downregulates insulin signaling in the muscle, although the mechanism remains unclear. Mice with Sepp1 deletion (Sepp1-/-) were found to be protected from diet induced obesity and insulin resistance. In a follow-up study, Sepp1-/- mice were found to be protected from the drop in serum adiponectin levels in response to a high-sucrose, high-fat diet, although adiponectin levels were not completely restored to wild-type. This implicates a partial, but direct role for Sepp1 in regulating adiponectin [88]. Because Sepp1 is associated with gluconeogenic enzymes and downregulation of the insulin signaling pathway, it was proposed as a potential drug target. In fact, the commonly prescribed glucose lowering drug, metformin, suppresses Sepp1 expression [92]. Further investigation demonstrated that metformin-induced inhibition of Sepp1 expression occurs in an AMPK and FoxO3 dependent pathway [93].

### 5.3. Selenoprotein M

Selenoprotein M (SelM) is localized to the endoplasmic reticulum (ER) and is thought to participate in thiol-disulfide exchange through its thioredoxin-like domain [94]. *In vitro*, SelM has been shown to regulate calcium signaling and protect against oxidative stress [95]. Because of its high expression levels in the brain, it was initially hypothesized that SelM offered neuroprotective properties. However, no deficits in learning and memory were observed in SelM knockout (SelM-/-) mice under a Se adequate diet [96]. Interestingly, SelM deletion in mice results in adult-onset body weight gain and increased adiposity, suggesting SelM may play a role in obesity. Immunohistochemistry further supported this hypothesis, as SelM was revealed to be highly expressed in the paraventricular nucleus and the arcuate nucleus of the hypothalamus, regions that are implicated in energy homeostasis. The arcuate nucleus contains neurons expressing the leptin receptor, which is activated by the adipocyte-derived peptide, leptin [97]. The downstream effects of the leptin receptor are carried out via the Jak2-Stat3 pathway. Leptin resistance in the hypothalamus leads to metabolic disease. Although a direct mechanistic relationship remains to be tested, SelM may play a role in energy metabolism through regulating leptin signaling. Whole body SelM deletion in mice results in elevated circulating leptin levels and diminished phosphorylated Stat3 levels in the hypothalamus, which are indicative of leptin resistance [96]. Furthermore, ER stress has been implicated in hypothalamic leptin resistance [97]. As SelM is an ER-resident protein, there is a possibility SelM may promote leptin signaling by protecting against ER stress. Currently, it is unknown whether SelM contributes to human obesity. Given the possibility that SelM may promote leptin signaling by mitigating ER stress, further investigation into this relationship is warranted.

### 5.4. Iodothryonine Deiodinase 2

Low Se levels have been associated with thyroid disorders such as goiter [98,99]. Moreover, thyroid hormones exert strong effects on obesity. The primary thyroid hormone in the bloodstream is l-3, 3′, 5, 5′ tetraiodothyronine or thyroxine (T4), a pro-hormone with four iodines and a long half-life. To become biologically active, one iodine is removed from T4, producing l-3, 5, 3′ triiodothyronine (T3). Thyroid hormone deiodination is catalyzed by a selenoprotein family, the iodothyronine deiodinases (Dio). Dio1 and Dio2 mostly convert T4 into active T3, and Dio3 converts T4 into inactive reverse T3 (rT3), or T3 into T2, leading to either inactivation or degradation of thyroid hormone. Local deiodination via Dio enzymes allow tight, controlled regulation of thyroid hormone levels [100].

Thyroid hormones are known for the regulation of metabolism and basal metabolic rate via regulation of energy expenditure, thus tight control of thyroid hormone levels can dictate effects on energy balance [101,102]. Diet-induced obesity in male mice has also been demonstrated to depend on Dio2 activity in the anterior pituitary activated by the c-Jun N-terminal kinase (JNK) pathway, controlling TSH levels and consequent thyroid hormone-dependent energy expenditure [103]. Moreover, mice with targeted deletion of Dio2 in the pituitary have less body fat, despite maintaining their oxygen consumption normally [104].

Specifically, Dio2 controls adaptive thermogenesis induced by cold and by diet in the brown adipose tissue. Mice with targeted disruption of Dio2 (Dio2 KO) lack proper adaptive thermoregulation [105,106,107] and are more prone to high fat diet-induced obesity [108]. Male and female Dio2 KO mice are insulin resistant even on a normal chow diet, with increased gluconeogenesis, and accumulate triglycerides in the liver, despite not yet displaying significant weight gain. Dio2 activity also controls feeding at the arcuate nucleus of the hypothalamus via local control of T3 levels, which contributes further to energy balance [109,110]. Inability to properly activate thyroid hormone via Dio2 was linked to glucose intolerance through hepatic insulin resistance. Intriguingly, human Dio2 gene expression is inhibited by the heterodimerization of liver X-receptor (LXR) with the retinoid X-receptor (RXR) [111]. Dio2 regulation by a classic lipogenic transcription factor was observed in LXRα and LXRβ double KO mice, which ectopically express Dio2 in the liver [112], suggesting a role for Dio2 inhibition in hepatic lipid deposition and obesity.

### 5.5. Selenoprotein T

Selenoprotein T (SelT) was first identified *in silico*, using an algorithm to identify SECIS elements in the human dbEST [113]. Containing a thioredoxin-like fold, SelT was proposed to possess redox activity [114], but its precise function remains unknown. Bioinformatics analysis revealed SelT to be localized to the ER, possibly being trafficked to the plasma membrane [115]. SelT expression in mice appears to be highest during development, with SelT mRNA expression in most tissues decreasing in adulthood, except in endocrine tissues such as the thyroid, pituitary, testis, and thymus [116]. The pituitary adenylate cyclase activating polypeptide (PACAP) is a neuropeptide which increases cAMP through adenylate cyclase stimulation, having implications in a variety of cellular processes, including cell survival and secretory function. SelT was recently identified as a target of PACAP [117]. In differentiated PC-12 cells, SelT is necessary for PACAP-dependent neuroendocrine secretion by regulating intracellular Ca^2+^ levels. Many neuropeptides regulate energy homeostasis, such as NPY (neuropeptide Y) [118], Agrp (agouti-related peptide) [119], α-MSH (α-melanocyte stimulating hormone) [120], among others. Identifying the neuropeptides under SelT regulation could provide greater understanding of the connection between dietary Se and energy metabolism. Immunofluorescence demonstrated SelT to be highly expressed in the adult pancreatic β and δ-cells, the latter of which secretes somatostatin [121], indicating SelT may be involved in glucose homeostasis. Conditional knockout of SelT in the β-cell resulted in defective insulin secretion, suggesting SelT is critical to β-cell function. Studies in the glucose responsive murine β-cell line, MIN6, determined that PACAP-induced insulin secretion depends on SelT expression. This indicates that SelT may regulate blood glucose at multiple levels. Although SelT is expressed in other metabolic tissues such as the pituitary and thyroid [116], the function of SelT in these tissues is unknown.

### 5.6. Selenoprotein S

Like SelT, Selenoprotein S (SelS) was first identified *in silico*, and was shown to localize to the plasma membrane [1]. Functionally, SelS has implications in ER-associated degradation (ERAD) [122], inflammation [123], and the transport of multi-protein complexes [124]. In 2003, a novel protein, Tanis, was characterized as a glucose-regulated protein in *Psammomys obesus*, an animal model for T2D [125]. Tanis was found to be expressed in insulin-sensitive tissues such as adipose tissue, liver, and skeletal muscle. Through yeast two-hybrid screening, Tanis was found to interact with serum amyloid A, a family of proteins associated with the acute-phase inflammatory response, which is typically elevated in T2D patients. Potentially, Tanis acts as a receptor for serum amyloid A. Tanis was later identified to be a SelS homolog [1], leading to the hypothesis that SelS links inflammation to T2D. In support of this, a positive correlation between serum amyloid A levels and SelS expression in the skeletal muscle and adipose tissue of T2D patients was reported [126]. SelS appears to be dysregulated in the disease state, as insulin stimulation increases SelS mRNA expression in the adipocytes of T2D subjects but not healthy subjects. Conversely, a different study found subcutaneous adipocyte SelS mRNA expression to increase in response to insulin in both obese and lean subjects [127]. This study also failed to find a correlation between serum amyloid A and SelS expression. However, SelS expression was found to be higher in obese subjects, with increased subcutaneous SelS expression in obese subjects associated with BMI, sagittal diameter, serum HDL, triglycerides, insulin, and insulin resistance. Additionally, SelS polymorphisms were correlated with higher diastolic blood pressure and circulating insulin. These individuals were also at a higher risk for cardiovascular disease. Taken together, these studies support the role of SelS in metabolic disease.

While SelS has been associated with T2D, its role in metabolic disease remains unknown. One possibility is that SelS plays a protective role. For instance, SelS has been shown to be upregulated in the hepatoma-derived HepG2 cells in response to glucose deprivation [128], albeit the physiological relevance is debated, as the low glucose concentration tested was 2 mM, well below the range of normal blood glucose levels in humans. However, SelS was also found to increase in response to ER stress, while overexpression of SelS conferred protection against oxidative stress MIN6 cells. Thus SelS may play a protective role, counteracting oxidative stress in T2D development.

## 6. Selenium Metabolism in Metabolic Disease

Generation of the Scly knockout (Scly-/-) mouse model established a connection between Sec decomposition and metabolic syndrome for the first time. The Scly-/- mice develop a metabolic syndrome-like phenotype under a Se adequate diet, displaying hyperinsulinemia, increased body weight, dyslipidemia, and reduced glucose tolerance [129]. Under these conditions, hepatic selenoprotein levels are unchanged, suggesting that Scly may function in glucose metabolism in a role that is independent from its ability to regulate selenoproteins. However, a mechanistic link between Scly and glucose metabolism has yet to be determined. The metabolic phenotype in the Scly-/- mice is exacerbated under Se deficient conditions [129], strengthening the hypothesis that Scly is involved in Se recycling during Se deficiency. Not surprisingly, likely due to the inability to recycle Se, Scly-/- mice have diminished hepatic expression of GPx1 and SelS, as well as diminished serum Sepp1. It is interesting to note that these selenoproteins have been implicated in metabolic disease. A more comprehensive analysis of the altered selenoprotein expression in Scly-/- mice may provide a better understanding of the selenoproteins that are influenced in metabolic syndrome.

High fat diet studies demonstrated that Scly-/- mice are more susceptible to diet-induced obesity than their wild-type counterparts [130]. Because the high fat diet given to the mice consisted of an adequate Se supply, it is not surprising that Scly-/- mice and wild-type mice on a high fat diet had similar hepatic selenoprotein profiles. Intriguingly, serum Sepp1 levels were elevated in Scly-/- mice, possibly indicating increased gluconeogenesis. This strengthens the notion that Se and carbohydrate metabolic pathways are interconnected. Investigation of TCA cycle parameters revealed that Scly-/- mice fed a high fat diet have elevated levels of pyruvate, pyruvate dehydrogenase, the gluconeogenic enzyme, pyruvate carboxylase, and the lipogenesis promoting acetyl-CoA carboxylase. Additionally, citrate synthase activity was increased in Scly-/- mice. These findings suggest that in addition to carbohydrate metabolism, Se metabolism also regulates lipogenic pathways.

## 7. Sex Differences in Se and Metabolic Disease

Sex differences in Se uptake and selenoprotein expression patterns have been described and are reviewed elsewhere [131]. Given the sexual dimorphism in Se regulation, it is unsurprising that human clinical trials hint at the possibility that the relationship between Se and T2D is sex-specific. In the NPC trial, the increased T2D risk in response to Se supplementation was limited to males [12], although one limitation of this study is that females were severely underrepresented, comprising only 25% of the subjects. Nevertheless, the NHANES III, found a correlation between T2D risk and high Se in males, but not females [10], a finding which was supported by NHANES 2003–2004 [11]. In contrast, but still supporting the hypothesis that the Se and T2D interrelationship is sex-specific, Akbaraly *et al*. [7] reported a correlation between lower baseline Se and T2D incidence among elderly French men, but not women. Understanding sex differences in the biological function and regulation of selenoproteins may explain the sexually dimorphic results of Se and T2D. Unfortunately, studies involving sex differences in the contribution of individual selenoproteins to metabolic disease are limited. This section will highlight some of the known sex differences reported in selenoprotein regulation of energy metabolism.

GPx1 polymorphisms were correlated with increased MetS incidence in Japanese men, but not women [84]. The initial studies that investigated obesity and hyperinsulinemia in GPx1 overexpressing mice only utilized male mice, thus it is unknown whether the effect of GPx1 overexpression in mice is sex-specific [76,77]. Serum Sepp1 levels were elevated in diabetic men and women compared to healthy subjects [85]. However, subsequent studies investigating Sepp1 and insulin resistance involved female mice. Whether Sepp1 directly induces insulin resistance through AMPK in male mice is unknown. In the same study, Sepp1 deficiency was found to produce an obesity resistant phenotype in male mice. Female mice were left out due to inconsistencies in the results. Both male and female SelM-/- mice demonstrated an increase in body weight and adiposity when compared to wild-type mice [96]. However, the increase in circulating serum leptin was limited to male SelM-/- mice, suggesting that SelM regulation of leptin signaling is sex-specific. Moreover, since both male and female SelM-/- mice develop obesity, the implication is that the development of obesity in these mice occurs through sex-specific pathways. Although sex differences in the association between SelS and metabolic diseases have not yet been described, there are sex differences in the amount of Se necessary to reach maximal murine hepatic SelS expression [132]. This discrepancy in SelS expression may contribute to the differences observed in the effectiveness of Se supplementation in a model of septic shock. We must not exclude the possibility that the sex differences in the regulation of SelS expression might result in sex-specific outcomes in metabolic disease.

The studies in Scly-/- mice were conducted in males as it was observed that female Scly-/- tended to display a mild metabolic phenotype, whereas the differences in males were more pronounced [129]. A plausible explanation is that the Se metabolic pathway does not interfere with energy metabolism in females. Recent evidence from mice with combined Scly and Sepp1 deletion (Scly-/-Sepp1-/-) demonstrates that female mice are less dependent on the Se recycling pathway for neurological function, as female Scly-/-Sepp1-/- mice do not exhibit the neurological deficits reported in male Scly-/-Sepp1-/- mice [64]. As the primary Se source for the brain and testes, Sepp1 is particularly critical for these tissues, supplying Se via the ApoER2 receptor [133,134]. Absence of either Sepp1 or ApoER2 results in a similar phenotype, consisting of behavioral deficits and male infertility. Strikingly, castration of male Scly-/-Sepp1-/- mice was shown to attenuate the neurological dysfunction present in uncastrated mice, offering a novel representation of the competition between the brain and testes for Se supply [135]. In the context of metabolic disease, it is conceivable that sequestration of available Se by the testes occurs at the expense of metabolic tissues, leading to altered glucose homeostasis in males.

## 8. Final Remarks

Se undoubtedly has numerous benefits to human health, with implications in T2D [15], cancer [136], male fertility [137], and neurological function [138], among others. However, the association of high Se intake and T2D in human epidemiological studies and clinical trials raises concerns regarding the practicality of Se supplements. Studies in animal models have been valuable in understanding that the effects of Se intake on T2D are dose-dependent, and perhaps plays contrasting roles in the diseased *versus* non-diseased state. Animal studies have also offered mechanistic insight, identifying potential links between Se and T2D, namely oxidative stress and inflammation. In addition, because the effects of Se occur through the enzymatic actions of selenoenzymes, the individual selenoproteins (GPx1, Sepp1, SelM, Dio2, SelT, SelS) that have been connected to T2D provide important information on the details of Se in T2D. Moreover, the Se metabolic pathway has been suggested to influence carbohydrate and lipid metabolism, strengthening the idea that Se does in fact play a role in T2D. However, the aforementioned sexually dimorphic association between Se and metabolic diseases reveals the complexity of Se processes. In order to reduce potentially undesirable effects of Se supplementation, and to further improve dietary guidelines for Se, improved understanding of Se metabolism and selenoproteins in both male and female subjects is essential.

## Abbreviations

The following abbreviations are used in this manuscript:

### Abbreviations

α-MSH: α-melanocyte stimulating hormone
Agrp: Agouti-related peptide
ARE: Antioxidant response element
Cth: Cystathione γ-lyase
Cys: Cysteine
Dio: Iodothyronine deiodinases
EFSec: Sec-specific elongation factor
ER: Endoplasmic reticulum
ERAD: ER-associated degradation
GPx1: Glutathione peroxidase 1
GPx4: Glutathione peroxidase 4
IL: Interleukin
JNK: c-Jun N-terminal kinase
LXR: Liver X-receptor
Met: Methionine
NF-κB: Nuclear factor-kappaB
NMD: nonsense-mediated decay
NPY: Neuropeptide Y
PACAP: Pituitary adenylate cyclase
PDX1: pancreatic duodenal homeobox-1
PSTK: Phosphoseryl-tRNA kinase
PTEN: Phosphatase and tensin homolog
PTP: Protein tyrosine phosphatase
rT3: Reverse T3
RXR: Retinoid X-receptor
SBP2: SECIS binding protein 2
Scly: Selenocysteine lyase
Se: Selenium
Sec: Selenocysteine
Sec-tRNA[Ser]Sec: Sec tRNA
SECIS: Sec insertion sequence
SelM: Selenoprotein M
Sepp1: Selenoprotein P
SelS: Selenoprotein S
SelT: Selenoprotein T
SeMet: Selenomethionine
SPS2: Selenophosphate synthetase 2
T2D: Type 2 diabetes
T3: l-3, 5, 3′ triiodothyronine
T4: thyroxine or 5, 5′ tetraiodothyronine
TNF: Tumor necrosis factor
TrxR1: Thioredoxin reductase 1
TrxR3: Thioredoxin reductase 3
